# Chemical Fingerprint Analysis and Content Determination of Horned Gallnut and Bellied Gallnut in Galla Chinensis

**DOI:** 10.1155/2023/8849341

**Published:** 2023-12-31

**Authors:** Xiaomei Long, Shuang Guo, Jianxing Gu, Lijun Zhang, Haipeng Liu, Yuan Fan, Weibo Wen

**Affiliations:** ^1^Yunnan University of Chinese Medicine, Kunming 650500, Yunnan, China; ^2^The Second Affiliated Hospital of Yunnan University of Chinese Medicine, Kunming 650041, Yunnan, China; ^3^The First Affiliated Hospital of Yunnan University of Chinese Medicine, Kunming 650021, Yunnan, China

## Abstract

To establish an objective and comprehensive methodology to analyse the connections and differences between horned gallnut (HG) and bellied gallnut (BG) in Galla Chinensis (GC). The HPLC fingerprints from 15 batches of HG and 15 batches of BG were performed, and chemometric analysis including similarity analysis (SA), hierarchical clustering analysis (HCA), principal component analysis (PCA), and orthogonal partial least squares discrimination analysis (OPLS-DA) was also set up. The results showed that the similarity of all batch samples was more than 0.9. In fingerprint analysis, 8 distinct common peaks were detected, among which gallic acid (GA), 1,3,6-tri-O-galloyl-*β*-D-glucose (TGG), and 1,2,3,4,6-O-galloyl-D-glucose (PGG) were identified by comparing with the standard compounds. Meanwhile, samples were clearly grouped into two classifications corresponding to HG and BG. This study demonstrated that HPLC fingerprints coupled with chemometric analysis could be applied to discriminate HG and BG and evaluate the qualities of HG and BG rapidly, which provided a certain experimental basis for the selection of GC raw materials and subsequent use.

## 1. Introduction

Galla Chinensis (GC), also known as *wubeizi* in Chinese, is a gall caused by the aphids on the *Rhus* leaves of the *Anacardiaceae* family (mainly *Rhus chinensis* Mill, *Rhus potaninii* Maxim, and *Rhus punjabensis*). These three original plants are mainly distributed in China, Japan, Korea, and other regions at an altitude range of 350–2700 m [1]. In the complex process of GC formation, the presence of the gall aphid and these three host plants are indispensable, which is the main reason why relatively few countries produce GC. It was first recorded as a medicine in the ancient Chinese medicine book *Bencao Shiyi* which was written in the Tang Dynasty and then was recorded for the treatment of lung deficiency, lung heat, phlegm cough, diarrhea, night sweat, bloody stool, hemorrhoid, and traumatic hemorrhage in Compendium of Materia Medica, an ancient Chinese medical book that was written by Li Shizhen in the Ming Dynasty [1, 2]. GC contains about 50–70% hydrolysable tannins which have a variety of biological effects, including antioxidant, antibacterial, anticancer, and antiviral, and is widely used in medicine, commercial inks, leather tanning, and chemicals, of which in leather tanning, GC has a softening and toughening effect [3–8]. GC is mainly distributed in most parts of China (except Xinjiang and Qinghai) and other East Asian countries, of which China accounts for more than 95% of the world's total production and has long-term been exported to Europe, America, Japan, and other developed countries [9].

Based on shape, GC can be categorized into two groups: horned gallnut (HG) and bellied gallnut (BG), also known as *jiaobei* and *dubei* in Chinese. Although the output of HG is higher than that of BG, the quality of BG is better than that of HG [9, 10]. In the measurement of wall thickness, it was found that the wall thickness of BG was significantly greater than that of HG, giving BG a firmer appearance, which is probably the main reason why traditional BG is superior to HG [10]. The GC formed on the leaves of *Rhus chinensis* Mill is called HG, and the acquisition time is mostly concentrated in September to October, while the GC formed on the leaves of *Rhus potaninii* Maxim and *Rhus punjabensis* J. L. Stewart ex Brandis is called BG, and the acquisition time is centered around May to June [1]. According to Chinese Pharmacopoeia (2020 edition), HG is rhomboid with irregular obtuse-angled branches and more prominent pilose, while BG is oblong or fusiform without bulge or branches. According to statistics, GC can be produced in many provinces of China, of which Guizhou, Sichuan, Hubei, Hunan, Shanxi, and Yunnan account for more than 90% of the country's total production [11, 12]. The HG is mainly distributed in the south of the Changjiang River, accounting for about 75% of China's total production, while the BG is mainly distributed in the north of the Changjiang River, accounting for about 20% of the total production [13]. Although HG and BG are very different in appearance and formation process, HG and BG are collectively known as GC, and there is no clear classification in use. Traditional Chinese medicine generally possesses complex compositions, and the formation and content of internal chemical composition are influenced by a variety of factors. The choice of raw materials also affects the composition of the extract and may change its chemical properties and biological activity [14]. Therefore, this study will investigate the differences and connections of HG and BG in their internal chemical composition, providing a certain experimental basis for the subsequent use and the selection of raw materials of GC. At present, there are fewer studies on the chemical fingerprinting of GC, most commonly on the gall parasitic process of GC, while the simultaneous study of HG and BG chemical fingerprints has not been reported [15, 16].

Chromatography fingerprint is a comprehensive and quantifiable analysis method that can elucidate the complexity and relevance of components [17]. The most commonly and conveniently used detection methods for fingerprint establishment are chromatography and spectrometry, among which high-performance liquid chromatography (HPLC) has the advantages of high separation, fast analysis speed, good selectivity, and wide application range [18–20]. HPLC is based on the principle that substances reach equilibrium through multiple partitioning between two liquid phases and uses the difference in partition coefficients of the components in the two phases for separation, preparation, and collection. It is widely used for the analysis and separation of various components. With the development of computer software, chemometric analysis is becoming more and more important in the discrimination of different Chinese medicine [21, 22]. Chemometric analysis can rapidly and accurately differentiate samples that contain virtually identical compounds by processing HPLC fingerprint data. Therefore, the method could be chosen to study the differences and connections of chemical composition between the HG and BG, which could develop the medicinal value and expand its applications, providing a wider and more effective choice of raw materials.

In this study, the chromatography fingerprint combined with chemometric analysis, including similarity analysis (SA), hierarchical clustering analysis (HCA), principal component analysis (PCA), and orthogonal partial least squares discrimination analysis (OPLS-DA), was developed to evaluate the differences and connections of chemical composition between HG and BG. In addition, the contents of gallic acid (GA), 1,3,6-tri-O-galloyl-*β*-D-glucose (TGG), and 1,2,3,4,6-O-galloyl-D-glucose (PGG) in 15 batches of each category were determined simultaneously by HPLC.

## 2. Materials and Methods

### 2.1. Materials and Reagents

**Figure 1:**
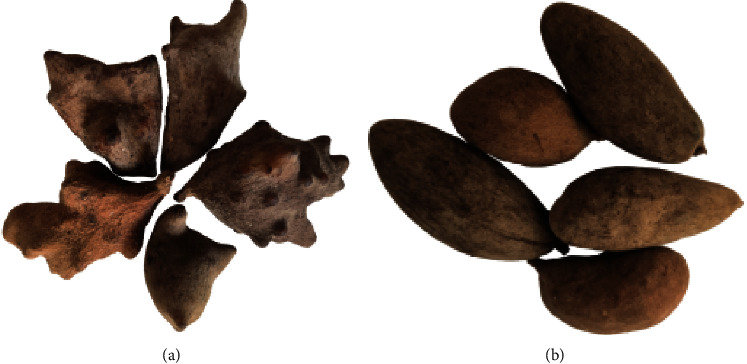
Morphology of (a) horned gallnut (HG) and (b) bellied gallnut (BG).

A total of 15 batches of HG (HG210601–HG2106015) and 15 batches of BG (BG210601–BG2106015), named HG1–HG15 and BG1–BG15, respectively, were collected from Jiangsu Province, China. According to their morphological characteristics, all voucher specimens were taxonomically identified by Associate Professor Jie Zhang (Teaching and Research Department of Chinese Medicine Identification, Yunnan University of Chinese Medicine). The morphology can be found in Figure 1.

Methanol (chromatography pure), methanol (analytical pure), and phosphoric acid (analytical pure) were purchased from Yunnan Danchi Trading Co., Ltd. (Kunming, China). GA (batch no.: wkq16081904, purity ≥98%) was obtained from Sichuan Weikeqi Biotechnology Co., Ltd. (Chengdu, China). TGG (batch no.: CFS201802, purity ≥98%) was obtained from Wuhan Tianzhi Biotechnology Co., Ltd. (Wuhan, China), and PGG (batch no.: PRF21060701, purity ≥98%) was purchased from Chengdu Prefa Technology Development Co. Ltd. (Chengdu, China).

### 2.2. Instruments and Equipment

The HPLC analysis was operated on Agilent 1200 high-performance liquid chromatography with an Agilent Zorbax C18 analytical column (150 × 4.6 mm, 5 *μ*m) (Agilent, Santa Clara, CA, USA). The D2KW-D electric thermostatic water bath was purchased from Shanghai Ailang Instrument Co., Ltd. (Shanghai, China), and the rotary evaporator was obtained from Shanghai Liangyi Scientific Instrument Co., Ltd. (Shanghai, China). QUINTIX35-1CN type electronic balance was purchased from Sartorius Scientific Instruments Co., Ltd. (Beijing, China), and the FA1004N type electronic balance was purchased from Shanghai Jinghai Instruments Co., Ltd. (Shanghai, China). DFY-600 swinging high-speed universal pulverizer was obtained from Yongkang Type Sufeng Industry and Trade Co., Ltd. (Yongkang, China).

### 2.3. HPLC Instrumentation and Chromatographic Conditions

HPLC fingerprint analysis was operated on an Agilent 1200 high-performance liquid chromatograph (Agilent, Santa Clara, CA, USA), and all samples were separated on an Agilent Zorbax C18 column (150 × 4.6 mm, 5 *μ*m, Agilent, Santa Clara, CA, USA). The mobile phase was composed of 0.1% phosphoric acid-water (A) and methanol (B). The condition used for the gradient program was developed as follows: 0–6 min, 5–7% B; 6–15 min, 7–25% B; 15–20 min, 25–28% B; 20–35 min, 28–33% B; 35–43 min, 33-34% B; 43–50 min, 34-34% B; 50–60 min, 34-35% B; 60–62 min, 35-35% B; 62–65 min, 35−5% B; and 65–75 min, 5-5% B. The flow rate was 1.0 mL/min, and the column temperature was 25°C. The detection wavelength was set at 280 nm, and the injection volume of each sample and standard solution was 10 *μ*L.

### 2.4. Preparation of the Sample and Standard Solutions

*Preparation of Standard Solutions*. The reference compounds were accurately weighed and dissolved in methanol to prepare the stock solutions. Their concentrations were as follows: GA (1.12 mg/mL), TGG (0.88 mg/mL), and PGG (1.78 mg/mL). All the solutions were stored in the refrigerator at 4°C before use.

*Preparation of Sample Solutions*. 15 batches of each category were cracked, and then the gall wasps in the shell were removed, powdered, and passed through an eighty-mesh sieve. 0.5 g of powder that was accurately weighed was refluxed with 20 mL of water at 90°C for 2.5 hours. The extraction was filtrated and the residue was washed with water. The filtrate was collected and transferred to a rotary evaporator to recover the solution water at 50°C. Methanol was added to dissolve and transferred to a 50 ml volumetric flask to volume. After filtrating through a 0.45 *μ*m membrane, 10 *μ*L of the obtained solution was injected into the HPLC system for analysis.

### 2.5. Software Methods

“Similarity Evaluation System for Chromatography Fingerprint of Traditional Chinese Medicine” (version 2012A, Chinese Pharmacopoeia Commission, Beijing, China) was used to establish the fingerprint and analyse the similarity by importing the chromatograms of 15 batches of HG and 15 batches of BG in AIA format. The HCA, PCA, and OPLS-DA were performed by SIMCA-P14.1 software (Umetrics, Umea, Sweden). Repeated measurement analysis of variance was performed using SPSS 26.0 (version 26.0, SPSS Inc., Chicago, IL, USA).

## 3. Results and Discussion

### 3.1. Validation of Analytical Method

#### 3.1.1. Precision, Stability, and Repeatability

According to the guidelines of the methodology, the system precision, stability, and repeatability were determined to verify the feasibility of the HPLC fingerprint method.

In order to test the precision of the method, the same sample was continuously measured six times. The stability experiment was carried out by measuring the same sample in 0, 4, 8, 12, 16, 20, and 24 h, respectively. To confirm the repeatability of the method, six replicate samples were prepared by using the same sample preparation procedure. The relative standard deviations (RSDs) of the relative retention time and relative peak area were calculated separately. The results are shown in Table 1, indicating that the analytical method was repeatable, the sample was stable, and the instrument had good precision.

### 3.2. Chromatography Fingerprint Analysis of Samples

**Figure 2:**
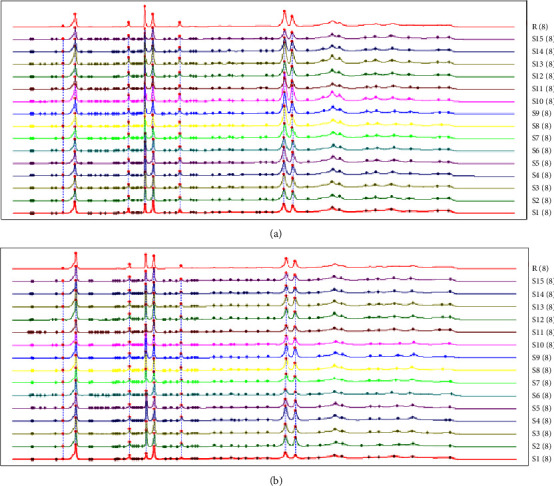
HPLC fingerprints of 15 batches of HG (a) and 15 batches of BG (b). The *X*-axis represents the retention time (t/min) and the *Y*-axis represents the response value (mAU).

**Figure 3:**
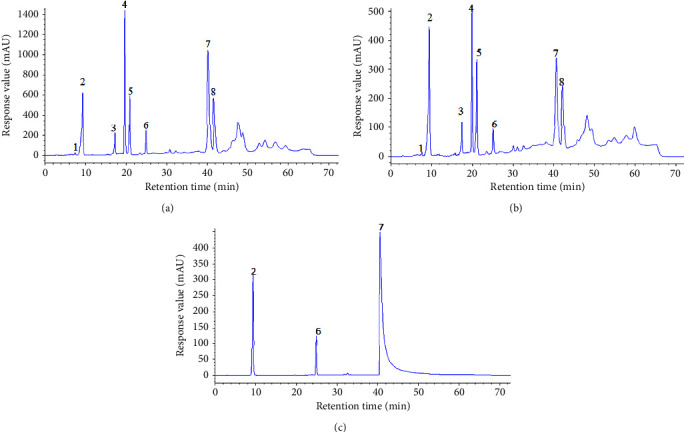
Representative chromatograms of HG (a) and BG (b), and HPLC chromatogram of mixed reference solution (c). Peaks 2, 6, and 7 were GA, TGG, and PGG, respectively. The *X*-axis represents the retention time (t/min). The *Y*-axis represents the response value (mAU). The peaks 1–8 were the main common peaks.

The HPLC fingerprints from 15 batches of HG and 15 batches of BG were established by Similarity Evaluation System for Chromatography Fingerprint of Traditional Chinese Medicine (version 2012A, Chinese Pharmacopoeia Commission, Beijing, China). Peaks that were existed in all sample chromatograms with reasonable heights and good resolutions were assigned as “common peak.” The time window was set to 0.1 s, and the calibration method was multipoint calibration. The reference chromatogram fingerprint was generated by using the average method. As shown in Figures 2 and 3, there were 8 distinct common peaks in the HPLC fingerprints, three of which (peaks 2, 6, and 7) were identified as GA, TGG, and PGG, respectively, by comparing retention times with the standard compounds.

The similarity of chromatographic fingerprint data is indicated by the correlation coefficient. The value of the correlation coefficient is close to 1.0, indicating that the different samples there have high similarity. On the contrary, a low correlation coefficient indicates a poor mathematical quality for identifying the relationship between different samples. As shown in Table 2, the similarity of the samples between HG and BG was larger than 0.983 and 0.961, respectively, indicating that HG and BG had good similarity and shared similar chemical components. The results confirmed that the fingerprints established in this study were reliable in assessing the quality of HG and BG. However, similarity analysis (SA) could not give more information for the connections and differences between HG and BG. Therefore, in order to analyse the inner quality of HG and BG more precisely, it is necessary to perform subsequent chemometric analysis, including hierarchical clustering analysis (HCA), principal component analysis (PCA), and orthogonal partial least squares discrimination analysis (OPLS-DA).

### 3.3. Hierarchical Cluster Analysis of Samples (HCA)

**Figure 4:**
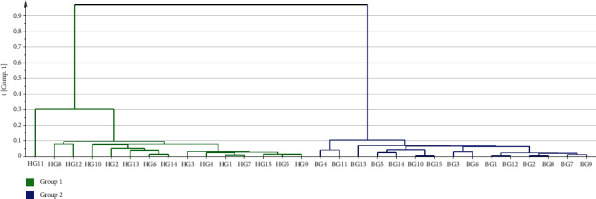
Dendrogram of HCA.

To assess the connections and differences between HG and BG, the HCA was performed by SIMCA-P14.1 (Umetrics, Umea, Sweden), and the Euclidean distance was used to measure the closeness between the samples. HCA is a multivariate analysis method that displays complex raw data in a visual form and provides classification information for test samples [23]. In this study, the relative peak areas of 8 common peaks from the 15 batches of HG and 15 batches of BG were used to form a matrix. As shown in Figure 4, the tested samples could be approximately categorized into two groups. The left of the dendrogram consisted of HG samples, whereas the BG samples were distributed on the right. From the results of cluster analysis, there were obvious differences between HG and BG, which might be related to the different formation processes of HG and BG.

### 3.4. Principal Component Analysis of Samples (PCA)

**Figure 5:**
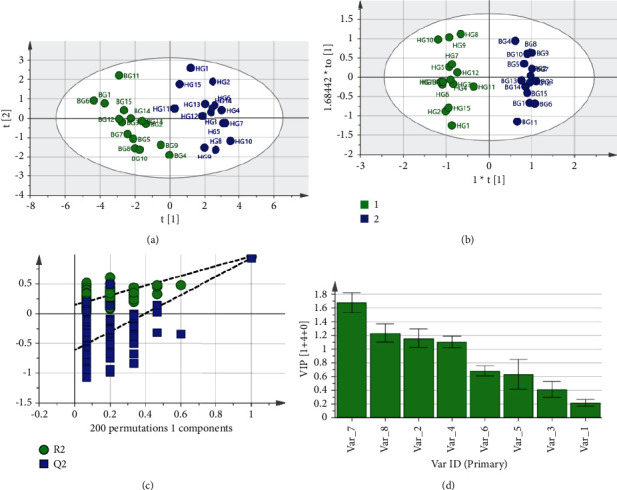
PCA score scatter plot (a), OPLS-DA score scatter plot (b), OPLS-DA model replacement verification (c), and VIP plot (d) of HG and BG.

PCA was required to assemble the original variables into a new set to further explore the homogeneity and quality of HG and BG. PCA is a useful approach to efficiently reduce the original high-dimension data into low-dimension data without much information loss and can be used to investigate the interrelationships between multiple variables [24]. The relative peak areas of 8 common peaks from 15 batches of HG and 15 batches of BG were imported into the SIMCA-P software to obtain the scores and loadings of multivariate analysis. As shown in the score plot of PCA (Figure 5(a)), the distribution distances of the samples represented the similarities and differences between these samples. Obviously, all samples were clearly grouped into two classifications corresponding to HG and BG, which reflected the differences in the chemical profiles between the HG and BG. In conclusion, PCA could be used to discriminate the HG and BG. The result of PCA was similar to that of HCA.

### 3.5. Orthogonal Partial Least Squares Discriminant Analysis of Samples (OPLS-DA)

OPLS-DA is a supervised model in which the users give the identity of each group sample in order to attain maximum variance of the groups in the hyperspace. It divides the systematic variation in the *X* matrix into two distinct parts: *Y*-predictive block and *Y*-orthogonal (*Y*-uncorrelated) block to improve the quality of the model [25]. Three main parameters *R*^2^*X*, *R*^2^*Y*, and *Q*^2^ are often used to assess the performance of the model. If the values of *R*^2^*X* and *R*^2^*Y* are close to 1.0, this suggests that the method is very suitable. Generally, the value of *Q*^2^ greater than 0.5 is acceptable, and the difference between *R*^2^ and *Q*^2^ values should be less than 0.3. In this research, OPLS-DA was conducted to obtain three important parameters: the diagram of the score scatter plot, the diagram of the OPLS-DA model replacement verification, and the variable importance plot (VIP). The results are shown in Figures 5(b)–5(d).

To further confirm whether there were differences between HG and BG and identify the characteristic components which have a significant influence on the chemical profiling of them, OPLS-DA was established with SIMCA-P14.1. The results are shown in Figure 5(b). The values of *R*^2^*Y* and *Q*^2^ were 0.996 and 0.965, respectively, indicating that the model had good stability and predictability. The OPLS-DA score scatter plot showed that all samples could be classified into two groups, corresponding to HG and BG. The OPLS-DA model could efficiently differentiate between HG and BG. The results were consistent with HCA and PCA, suggesting that these three methods could be used to distinguish between HG and BG. In addition, in order to further test the validity of the model and the differences between the samples, a permutation test of 200 iterations of the established OPLS-DA model was performed. As shown in Figure 5(c), the *R*^2^*Y* and *Q*^2^*Y* values of the original OPLS-DA models were still significantly higher than the corresponding values of the permuted models. In addition, the *R*^2^*Y* intercept and *Q*^2^*Y* intercept for the established OPLS-DA models were less than 0.3 and 0.05, respectively. These results showed the validity of the model.

The variable importance plot (VIP) produced by OPLS-DA gave a good reference for identifying variables that had a significant impact on the classification. The VIP value is usually used to explain the contribution of a variable to the model, and the variable that VIP value is greater than 1 is considered to be important [26]. As shown in Figure 5(d), four components with VIP >1 including chromatography peaks 7 (PGG), 8, 2 (GA), and 4 were selected as potential chemical markers, which could be used to differentiate between HG and BG. Peaks 8 and 2 could not be identified because of experimental limitations, which also was the shortcoming of this experiment, and subsequent studies will be combined with mass spectrometry to help identify more compounds in HG and BG. PGG has shown strong biological and pharmacological activities in antiviral, anticancer, anti-inflammatory, antimicrobial, and antidiabetic [27]. There are diverse scientific reports on the biological and pharmacological activities of GA, focused on antioxidant, antimicrobial, anti-inflammatory, anticancer, cardioprotective, gastroprotective, and neuroprotective effects [28]. Our group has also related research on PGG and GA in the treatment of diabetes and found that PGG and GA could inhibit islet *β*-cell apoptosis in a high glucose state [29–31].

### 3.6. Quantitative Analysis of Samples

#### 3.6.1. Method Validation of Quantitative Analysis

*(1) Linearity, Limits of Detection (LOD), and Limit of Quantification (LOQ)*. A series of calibration curves were constructed between the peak areas (*Y*) and the concentrations of reference substances (*X*) in the investigated ranges. The limits of detection (LOD) were calculated with *S*/*N* = 3, and the limit of quantification (LOQ) was calculated with *S*/*N* = 10. The correlation coefficient values were more than 0.9993 at a series of gradient concentrations, which indicated that there was a satisfactory correlation between the concentrations and peak areas of the three compounds at a relatively wide range of concentrations. The results are shown in Table 3.

*(2) Precision*. The precision check was performed by continuously injecting 6 times according to the same chromatography conditions. The RSD values for the peak areas of GA, TGG, and PGG were 1.06%, 2.28%, and 1.34%, respectively, indicating that the instrument had good precision.

*(3) Stability*. Sample stability test was performed by running the same sample at 0, 4, 8, 12, 16, 20, and 24 h. The RSD values for the peak areas of GA, TGG, and PGG were 3.18%, 1.11%, and 1.24%, respectively, indicating that the test solution was stable within 24 h.

*(4) Repeatability*. To confirm the repeatability of the method, six replicate samples were prepared by using the same sample preparation procedure. The RSD values for the peak areas of GA, TGG, and PGG were 2.81%, 1.49%, and 3.78%, respectively, indicating that the method was reproducible.

*(5) Recovery*. In the recovery test, an appropriate amount of sample was weighed and spiked with a known amount of each standard compound. Then the sample was treated and analysed as previously described. The results showed that the average recovery of GA, TGG, and PGG was 100.13%, 98.04%, and 99.41%, and the RSDs were 1.39%, 1.82%, and 1.09%, respectively, which showed that the experimental method was suitable.

#### 3.6.2. Content Determination of Samples

**Figure 6:**
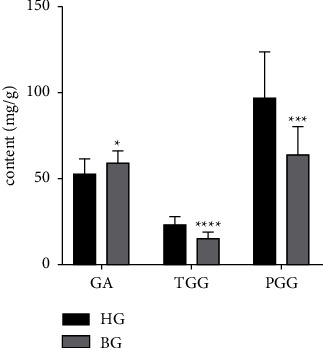
The contents of GA, TGG, and PGG in HG and BG (mg/g). Compared with the HG, the contents of GA, TGG, and PGG had significant difference (^*∗*^*p* < 0.05).

The established HPLC method was used to determine the contents of GA, TGG, and PGG in 15 batches of HG and 15 batches of BG. As shown in Figure 6 and Table 4, found that the content of TGG was the lowest in HG and BG compared with GA and PGG. GA and PGG were used as potential compounds to differentiate HG and BG. The GA content in BG was significantly higher than HG (*P* < 0.05), while the contents of TGG and PGG in BG were significantly lower than HG (*P* < 0.001). If only the difference in GA content between HG and BG were analysed, this result was consistent with the traditional belief that “bellied gallnut is superior and horned gallnut is inferior.” However, the evaluation of the quality of traditional Chinese medicine is not comprehensive if it is based only on a single component. Based on the results of OPLS-DA, PGG was identified as an important chemical marker for HG and BG, and its content was higher in HG than in BG. Therefore, the quality of HG and BG should be evaluated from multiple aspects, perspectives, and components rather than a single component, which provided some reference for the selection of GC raw materials.

## 4. Conclusions

This study is different from previous studies, which only focused on the HPLC fingerprint of GC or content determination of some compounds in HG or BG [8, 32]. In this study, we analysed the connections and differences between HG and BG by analyzing different batches of HG and BG by SA, HCA, PCA, OPLS-DA, and content determination of samples, which provided a certain experimental basis for the selection of GC raw materials and subsequent use. The results indicated that fingerprint combined with chemometric analysis was a powerful and practical method to objectively and rapidly differentiate between HG and BG. However, this paper only analysed the connections and differences between HG and BG from the perspective of chemical fingerprint analysis. In the follow-up, our group will further investigate HG and BG in more depth from metabolomics, transcriptomics, rat models, and clinical treatments so as to more comprehensively observe the differences between HG and BG.

## Figures and Tables

**Table 1:** The relative standard deviation (RSD) results of methodology validation.

Methodology	The range of relative retention time (%)	The range of relative peak area (%)
Precision (*n* = 6)	0.06%–0.21	0.52%–2.38
Stability (*n* = 7)	0.06%–0.12	2.38%–3.63
Repeatability (*n* = 6)	0.09%–0.14	1.66%–4.34

**Table 2:** The similarity values for 15 batches of HG and 15 batches of BG.

Sample	Similarity value	Sample	Similarity value
HG1	0.983	BG1	0.981
HG2	0.995	BG2	0.998
HG3	0.998	BG3	0.999
HG4	0.999	BG4	0.982
HG5	0.999	BG5	0.999
HG6	0.999	BG6	0.961
HG7	0.999	BG7	1.000
HG8	0.992	BG8	0.996
HG9	0.995	BG9	0.989
HG10	0.993	BG10	0.996
HG11	0.993	BG11	0.996
HG12	1.000	BG12	0.997
HG13	0.999	BG13	0.999
HG14	0.999	BG14	0.999
HG15	0.986	BG15	0.997

**Table 3:** Calibration curves, *R*^2^, linearity range, LOD, and LOQ of compounds.

Compounds	Calibration curves	*R* ^2^	Linearity range (mg/mL)	LOD (mg/mL)	LOQ (mg/mL)
GA	*y* = 14258*x* + 194.29	1.0000	0.186–1.87	0.019	0.063
TGG	*y* = 6353.2*x* + 77.962	1.0000	0.11–2.20	0.009	0.028
PGG	*y* = 26679*x* − 5121.8	0.9993	0.35–2.136	0.004	0.013

**Table 4:** The contents of GA, TGG, and PGG in HG and BG (*n* = 3) (mg/g).

Sample number	GA	TGG	PGG
HG1	45.20267587	16.85404963	62.09240741
HG2	46.07508324	20.50526685	78.03353667
HG3	47.60650029	22.88319288	89.60223495
HG4	48.07691603	24.05992509	93.49234997
HG5	48.35146776	24.59936798	99.79201989
HG6	47.33579742	22.02832397	82.70847814
HG7	47.94391667	24.47255384	94.04958485
HG8	52.43938052	23.40203652	119.9229399
HG9	62.93692153	25.28043071	126.7065456
HG10	69.30335706	34.24110487	161.6402321
HG11	50.83098635	19.30922285	69.61062366
HG12	52.0763051	22.68235019	94.23910951
HG13	73.86724501	31.11709972	125.2409693
HG14	49.9064056	23.7380618	86.68242137
HG15	47.92125118	16.96927669	62.38560367
BG1	58.7117329	10.83842463	49.12090271
BG2	59.5405199	17.98572097	68.69215803
BG3	63.20334789	14.72331461	64.77693505
BG4	58.63689403	20.77241339	94.80525367
BG5	58.58215475	16.25152154	64.89518547
BG6	58.41152213	9.791081461	42.47417589
BG7	60.28719797	15.74104635	64.81378706
BG8	61.12197208	16.5508544	76.93607553
BG9	58.95805969	20.50655431	84.43849806
BG10	49.61816904	13.92316245	63.78274054
BG11	44.62363686	7.823852996	37.4375987
BG12	77.26664019	17.21003043	76.71253363
BG13	63.08232275	19.13477294	70.97819793
BG14	53.08812658	14.34866573	56.8460572
BG15	58.61679445	14.02294007	58.73563421

## Data Availability

The data used to support the findings of this study are included in the article.
